# The Causation of Disease—Contagion

**Published:** 1871-12

**Authors:** Frank A. Ramsey

**Affiliations:** Knoxville, Tenn., Vice-President of the Tennessee Medical Society; President of the Medical Society of East Tennessee; Corresponding Member of the Gynæcological Society of Boston ; Local Secretary, etc., of the Anthropological Society of London


ATLANTA

### MEDICAL AND SURGICAL JOURNAL.

NEW SERIES.

VOL. IXB-DECEMBER, 1871.—NO. 9.

### ORIGINAL COMMUNICATIONS.

[Communicated by SWAN M. BURNETT, M.D., Secretary Medical Society East Tennessee.]

### THE CAUSATION OF DISEASE—CONTAGION.

BY FRANK A. RAMSEY, M.D., KNOXVILLE,

Vice-President of the Tennessee Medical Society; President of the Medical Society of East Tennessee; Corresponding Member of the Gynæcological Society of Boston; Local Secretary, etc., of the Anthropological Society of London.

*Read to the Medical Society of East Tennessee, for Knox County, December*, 1871.

"The true philosophy of the Science of Medicine is the knowledge of the causes of disease; or, if these causes be too subtle and refined for our gross senses, it is the knowledge of the several conditions external or internal to the body, which give those causes power. In the future history of medicine, we shall see men returning to the principles promulgated by its earliest founders. They will perceive that the treatment of the fully-formed disease is at the same time the most difficult and the least useful part of their most noble profession. They will learn to arrest the evil at the fountain-head, and not to dam the current swollen by a thousand tributaries." R. V. W.

"That is a great mission of the physician, to assuage pain, calm the perturbed mind, and heal the sick; but it is the greater mission of the mediciner to instruct others how to avoid sickness." ANON.

"Reasoning from single individual instances will generally deceive; but well-digested impartial observation upon masses of men can never lead to an erroneous conclusion." FERGUSSON.

T

In the Medico-Chirurgical Review for January, 1870, it is declared that the "old hypotheses on contagion have been overturned."

But the writer in the Review cannot ignore the mortifying necessity of connecting with the declaration the humbling confession, that "in spite, however, of the many positive results obtained by medical observation, chemistry and microscopy in the last few years, considerable obscurity still surrounds the whole question of the nature, origin and prevention of contagion."

The subject is one of grave importance, involving monetary interests to so large an extent as to have elicited the attention of legislative bodies and special conventions of great commercial countries, and almost anuually gives occasion to forensic force in the "Councils" and "Boards of Mayor and Aldermen" of municipalities.

"The vexed subject of quarantine" is but one of the inevitable troubles resulting from the unsettled or discordant views that are defended regarding contagion; yet it is the trouble that forces the "men of authority" to appeal to the labor and solicit the services of mediciners, thus coalescing and identifying scientific and national interests.

The great social, commercial and scientific interests involved justifies the introduction of contagion as a topic for consideration by the members of this special session of the Medical Society of East Tennessee.

The fact is established by scholars, that the ancients, earth's inhabitants during the earlier centuries of historic record, were familiar with the subject of contagion; but what hypotheses entertained in the then state of familiarity with the subject, that have been overturned during the very many years, constituting ages since, it is not within the scope of this lecture, or the capacity of this lecturer, to expose.

But the hypothesis embraced in the definition given by Cullen, and received very nearly a century ago, is also expressed in the definition made seventy years later by Dickson, of this country; and if this is an "old hypothesis," I question much the verity of an assertion from any source, that it has been overturned.

The employment of a word is often a source of difficulty in scientific investigations, and more frequently constitutes the entirety of originality—is all that is new in the assumptive consideration of important subjects by claimants of praise for having superseded preëntertained views, which are, in fact, but reënunciated, albeit reclothed, perhaps disfigured, certainly disguised. Thus, Cullen called contagion "effluvia;" Dickson expressed it as a "peculiar modification of matter;" and Holland affirmed it to be "a *materies morbi*—a material cause." But the very definite confession of want of knowledge conveyed by the employment of such simple words has been "overturned" recently by the introduction, in their stead, of "some living organism," "germinal matter," "embryonic cell," "living organic ferment," etc.

The use of words with equivocal or diversity of meaning gives latitude for the display of some learning, but seldom serves to enlarge the field for the application of practical knowledge. In this instance, the mediciner and the political economist will find themselves, doubtless, more capable in opposing "effluvia," or "a material cause," or "peculiar modification of matter," than in essaying search after "some living organism," or in effort to probe the reconditeness of "germinal matter," or in endeavoring to trace the successive steps in development of the "embryonic cell." On the one hand, there is a something assumed, it is true," but its entity not the less doubted because of uniform results of identical effects; on the other hand, a something with specific nature indicated by the designative employed, that arouses desires for and belief in the necessity of inquiry in a particular direction—weakening, for awhile at least, the practical advantages to be derived from any consideration of the subject.

Oersterlin, of Heidelberg, instructs his readers, that "the language of science ought to furnish us special words and expressions wherever we are in need; and each of these words or expressions should convey exactly what we wish to assert concerning the things designated by them, and should render perfectly clear to others what we intend to express. Every word must have its exact meaning, its precise and definite value; no expression ought to be obscure, or admit of being taken in more senses than one."

The study of first causes or the conditions in which a contagion originated is seldom permitted, and mediciners, in their honesty, are ready to acknowledge ignorance in this particular. For the present, they are content with the idea expressed, in 1819, by Richard Gratten, of England:

"In the course of those changes which take place without interruption amongst the various kinds of organized matter, products may and do occasionally arise, which never before existed, and which, when applied to the system, are capable of producing diseases of a character totally different from any hitherto observed. And if it be possible for any cause, or combination of causes, to occasion a new disease, why may not that disease be contagious, and be again called into existence independent of contagion by the same causes which first produced it? As yet the revealments of science have failed to overturn the affirmation, and the negative of the question remains to be accomplished. When it is, we must be reduced to the necessity of supposing that all contagious diseases were derived from Adam himself."

Almost any febrile, or epidemic, or endemic, or sporadic disease may, under appropriate circumstances, become capable of generating in and about the sick ones an efficient cause of disease; but, ordinarily, the efficiency of the cause, its own existence, is preserved only so long as the appropriate circumstances prevail. Indeed, under appropriate circumstances, human bodies in health, or apparently in health, may generate an efficient cause of disease not regenerated in those persons first affected by it, except they be placed under precisely the same circumstances, or relationships, that prevailed in the origin of thecause. A notable instance occurred in England many years ago, and is referred to by almost every systematic writer. Very many prisoners had been for a long time crowded together in a dungeon, without regard to comfort or cleanliness. The court sitting, the prisoners were brought out; and though not laboring under the disease themselves, such was the condition of their persons and of their clothing as to contaminate the atmosphere of the court-room, occasioning so large number of persons to sicken and die that the session received the designative by which it is yet referred to by lawyers and physicians—the "*Black Assizes.*"

In all such instances, the capacity of the cause is limited to the effects from its first impression, and the cause itself is destroyed with the disappearance of the relationships in which it had origin—being itself, whether material or not, wholly destitute of individuality or self-existence.

Observation has failed to detect the source of the causes which occasion diseases in the animal economy, and are regenerated in the midst of, and by the effects of, their production. These causes are contagions, each of which is self-existing, and has individuality in quality, requiring for dissemination and multiplication only animal economies, as the seedling requires properly adapted soil in which to develop its forces. But it is improbable that the source of their origin has any relationship with excessively high temperature, because very many years have passed without having furnished successful denial to the averment made by Fergusson—that "contagious fever, with the exception of the exanthemata, cannot exist in the torrid zone, nor anywhere else in a temperature amounting to eighty degrees Farhenheit, long continued. It is infallibly dissipated by it. The heat of the tropics is prolific of fevers, but never of contagious ones."

These causes are, Cullen said, "efflluvia from the body of a man under a particular disease, and exciting the same kind of disease in the body of the person to whom they are applied." These causes are, Dickson said, "peculiar modifications of matter given out by a diseased body, which possesses the characteristic power of generating in a healthy body, when brought to act upon it, a condition of disease similar to that which produced it."

In October, 1871, it was declared, through an authoritative medium, that "these maladies destroy annually a full fourth of the population of Great Britain—namely, diseases which take their origin in the admission into the economy of a material which reproduces itself largely therein, and, being given off therefrom, spreads disease to other individuals." Several years before that was written, another mediciner said, in a leading English medical journal, concerning a particular and widely-prevailing disease: "The Sanitary Commission have paid much attention to this subject in their late inquiry, and have expressed their decided conviction that contagion does not belong to it; in other words, that those sick or dead of it do not emit a material capable of communicating the disease to others." The beloved La Roche, in that monument to himself as a laborer, durable testimony to the capacity of American mediciners, and splendid gift to professional literature in the English language, "On Yellow Fever," employed "the word contagion to signify a poison, effluvium, or emanation generated by morbid secretion in the course of a distemper, and possessing the power of inducing a like morbid action in healthy bodies, whereby it is reproduced and indefinitely multiplied, whether by contact, near approach, or the medium of external bodies impregnated with it." In 1812, the editors of the New York Repository, the pioneer of medical journals in America, defended a definition making "contagion a poisonous material capable of exciting a peculiar disease, and emanating with that power from a body sick with that peculiar disease."

Here, then, is an explicit meaning attached to the term contagion—a meaning so very definite that it does not give opportunity for disquisition and argumentation to word-mongers and cavilers—a meaning established by long usage, and having precision enough to satisfy the most exacting votary to science.

Matter is a contagion only when it occasions morbid effects in a living animal economy, and in the midst of the effects, or resulting from them, is itself reproduced with all its original capacities. Matter may be a morbific cause, and occasion disease in a living animal economy; yet this alone is not enough to place it with the contagions. But, in the event of the animal economy, which has been diseased by the matter, communicating the disease to another animal economy, under relationships so changed as to preclude the probability, if, indeed, not the possibility of dependence upon conditions outside of the matter and the living animal economy, then truly is the matter entitled to the dignity of classification with the contagions; for "the true essential contagions act of themselves, independent of and unaided by the circumstances of climate, atmosphere, locality, quantity and accumulation."

The coördinating circumstances in which the morbific cause originated may, perhaps, be designated; but for the most part, it is the essential nature of the matter expressed in its capacity to propagate itself with or from the effects that it occasions, to which interest is attached. This is all that is comprised in the meaning that, by common consent, is given to the term contagion; and any disquisition or investigation regarding the subject, and made without intimate regard to this meaning, will subserve no good practicable purpose, but will only open an arena for wordy contention, and furnish opportunity for argumentative display, often resulting most perniciously to commerce, to social virtue, and to the health and lives of individuals.

Contagious matter is an entity without tangibleness, without detectable materiality; no chemical manipulation has exposed its reactions, no microscopic power has exhibited its size, and only on life-endowed tissues and systems is its real being demonstrated.

Lymph known to contain contagious matter has been accurately divided into three elements—corpuscles called "leucocytes," granulations not exceeding one-twentieth thousand of an inch in diameter, and a clear liquid in which they both float. Neither one nor the other of the elements has proved capable to produce the effects occasioned by the lymph in its constituent form. And experimental efforts concur in establishing the facts, essentially important, that contagious matter is indiffusible and insoluble; that each particle in itself represents the whole from which it has been separated; that a myriadth of contagious matter communicates disease as completely as would a larger, and perhaps more appreciable, size of the same matter. "Consequently, when disease is communicated from one to another through the air, the fact signifies simply that the contagious particles are wafted by currents from the diseased organs of the one to the other; and hence the question of mediate contagion, like that of direct contagion, must be one of chance—the chance of meeting in the medium with the contagious particles."

A contagious matter, in contact with an animal economy susceptible to its impressions, are the only factors engaged in propagating contagious disease.

Contagious particles may be placed in efficient contact with a healthy susceptible economy at the place occupied by the economy in disease, and producing contagious matter.

Contagious particles may be placed in efficient contact with a healthy susceptible economy at localities remote from that occupied by the economy in disease, and producing contagious matter.

Contagious particles may be placed in efficient contact with a healthy susceptible economy at a locality remote from or at that occupied by the economy in disease, and producing contagious matter, without regard to the lapse of time. However short or long a time has passed since the contagious matter was produced, it requires only contact with a susceptible economy for the exhibition of its disease occasioning capacity.

Contagious particles may be placed in efficient contact with a susceptible economy through inorganic and organic media—through fabrics, earth, air, and water.

Exhibitions that essentially belong to the disease occasioned by the contagious matter are more or less severe, according to the great or less susceptibility of the individual impressed by the contagious matter. And the impressed susceptibility is modified—indeed, in by far the largest number, is wholly destroyed—by the operation of the contagious matter, giving occasion to the expression of a rule, having exceptions, that a contagious disease is taken only once in a life-time.

The idea that contagion originates from filth, bad diet, crowding, etc., is ordinarily erroneous, though very generally believed, notwithstanding it was, a long while ago, denounced by a leading Glasgow mediciner, as "a notion belonging to fancies contrary to sound philosophy." Filth, bad diet, crowding, etc., are circumstances conducive to low health, and in themselves are, or give occasion to, morbific causes. But filth, bad diet, crowding, etc., in no degree increase the susceptibility of individuals to be impressed by contagious matter. They in no degree shorten or prolong the period of incubation—the time from the moment the matter is received by an economy to the moment when the effects occasioned by it become observable. They in no degree increase the violence or affect the duration of an attack, or modify it in its progress, in individual instances of contagious disease.

The proof of such propositions can only be furnished in the proper exercise of discrimination between cause and coincidence, by observers of disease. But it will be conceded, that concurrence of circumstances by no means constitutes the relation of cause and effect, and that it is altogether possible for concurrence of circumstances to be observed, yet without any necessary connection—not dependent, not interdependent, and not conspiring to the production of the same effects in progress of action upon the same economy, though each after its own manner, and without agreement in detail, may contribute to bring about entire disability of the vital machine to be further impressed or operated.

Contagious diseases, for the most part, can be traced in the succession of attack. All the members of one household presumptively receive the poison from the first one who sickened in the family, and visitors carry it away and propagate it in other houses at other localities. Individuals must sicken from contact with sick persons, or contact with the poison through a medium traceable, beyond all peradventure, to the sick person, and, in other and differently located houses, themselves be the occasion of yet others having the same disease, to establish its character to be contagious. The character must be proven in this way, or it will be nothing more than assumption to say that cases occurring at points remote from that at which it was known to prevail, were contaminated by contagious particles wafted through the air, floated in water, or borne on clothing. The sickening of physicians and nurses in even the scrupulously clean, well-ventilated, properly-constructed and located hospitals, is certainly evidence of morbific cause in and about the persons in wards; but if the physicians and nurses occupy beds, and pass through the successive stages of the ailment in rooms more or less remote from the hospital, without others contracting the disease, the essential proof of contagion is wanting.

Unless it is so evident that "he who runs may read," it is improper to ascribe contagious character to a disease whenever it can be shewn to be caused by conditions, circumstances, or matter not possessing features appertaining to contagion; or, in the stronger language of Dr. John Hastings—"Humanity demands that the idea of contagion should be eschewed by the profession in every epidemic disorder, unless it be so beyond the shadow of a doubt, since it calls forth the worst feelings of the human heart in its ungovernable terror, and causes even the mother to desert her dying child, and the sick to languish uncared for and shunned."

Relative slowness in extending generally attends the presence of contagious diseases; and all the members of a family who sicken with it presumptively contract it from the first one who was sick, and not from each other successively. Hence, succession in attack is properly accounted for by a difference in susceptibleness of each of the several members of the family. And though it is true that occasionally a contagious disease may be widespread, yet, in the absence of essential proof, the fact of a large number, or of several persons at different points of a comparatively-extended area of locality, being attacked simultaneously, coincidently, or with marked rapidity in succession, militates greatly against the contagious origin or nature of the disease.

The contagious character of disease is almost always susceptible of proof:

In its traceableness from the sick, perhaps from the beginning, certainly during its progress through the well to the production indefinitely of the same kind of sickness; and in its course and extension being entirely controllable by the exclusion or seclusion of the sick, and by the destruction or perfect purification of the substances and atmospheres that may have been employed by and about them; and in the efficiency of cordons and quarantines, preventing the well and sick from any possible contact.

I wish I could control Milton's power in argumentation, and Robert Hall's facile use of English words, to elaborate the thought and circumstances connected with the application of exclusion or seclusion of the sick, and the practice of cordons and quarantines. If I could, public opinion would henceforth hold medical men to a stricter sense of responsibility, and prevent hastily ascribing contagious character to any disease, much less to a disease that from wide extent in prevalence, and perhaps from heavy mortality, already has aroused the people, filled them with intense anxiety, almost dread, just verging on the limits of fortitude—requiring nothing more, indeed, to be thrown into the whirl of consternation than the announcement from medical men that the disease is contagious. There are but few of us here, perhaps, who have ever encountered a contagious disease occasioning so much of terror, and showing the worst side of human nature in all its nakedness; but just as few, perhaps, who have not seen a partial exposure of that worst side in epidemic and endemic sicknesses to which the suspicion of contagious character had been—ignorantly may be, culpably surely—permitted by medical men.

In the literature of the profession, there is abundant testimony of the depraved morality and crippled commerce resulting from quarantines; and in the medical journals of almost every decade are reports of individual instances of inhumanity, such as occurred in 1832, when a pestilence was prevailing in the world, and cases had presented in Air, in daily connection with Gervan, England. "A very few days after the first appearance of the pestilence at Air, the post-man was seized by this distemper at Gervan, and there abandoned, under the prevailing panic of contagion, by all but one poor and humble friend, who administered to him until he died, and afterwards got a coffin and buried his body. For these brave and generous offices of Christian and brotherly benevolence, his own recompense from his neighbors and townsmen was to drive himself from his own home and family, and compel him to wander until the Board of Health could assemble and provide a lodging for him remote from his own family, there to abide while the Board's alarm for contagion should last. About the same time, a medical man from Air, passing near the village of Slateford, discovered a poor woman lying on the roadside. Only two kind men could be induced to give assistance, while he hastened for medicines and the medical man of the district. On their return, it was with great difficulty anything could be done; the villagers, insane from terror of contagion, refused to approach the woman, or even furnish any means of restoring warmth to her body. The medical man and the two humane volunteers were assailed by a torrent of abuse and threats of personal violence, and even of burning the house in which they were with the sufferer, if they should not instantly remove her from Slateford. It was vain to attempt to reason with them on the inhumanity and absurdity of their conduct. The practitioner was overpowered by a multitude of voices of men and women repeating their threats, by which he was reluctantly forced to yield. She was permitted only a wheelbarrow, on which she was placed and removed a short distance, when a blacksmith's wife offered her accommodation; but the people were relentless, and forced the woman to be taken to Air, where she was admitted into hospital, but too far gone: under the disease, the fatigue and the inhumanity, she died in a very few hours."

The gentleman who contributed the facts for publication says: "It is in sorrow, not in anger against the delusion which is the sole cause of such enormities, and not against those deluded individuals who have been the misled and ignorant perpetrators, that I wish to bring the facts under general consideration."

Those instances are reported from England; similar ones have occurred in "the land of the free and the home of the brave," in America. In the Paris (France) Medical Gazette, January 9, 1833, is published an extract from a letter written from Watertown, N. Y., in which it is affirmed that "the doctrine regarding contagion has caused the people in the United States to treat those sick with the pestilence like wild beasts." Remarking upon the published extract, a writer in the leading English medical review uses strong language: "A dungeon and bread and water for the remainder of their lives would be but inadequate punishment for the mischief inflicted on society everywhere by persons who have, from interested motives, been active in propagating the most fiendish doctrines."

It has never, I believe, gone to record that like results have been exhibited in our own East Tennessee, under the same debasing, demoralizing panic of contagion. In the Knoxville sickness of 1838, very many of the citizens left parts of their own families and their neighbors, and in many instances only to die, themselves denied, away from their own hearth-stones, the care, attention and home sympathies which they had placed themselves beyond the opportunity to give, and as well to receive; for, having carried the seeds of disease and death away with them, they carried, too, the panic by the force of which they had fled from their own kin and neighbors, and drove from their own beds of sickness and of death the strangers upon whom they had forced themselves, and who "would have none of them." Under the panic of contagion, the people of the country not only refrained from trading in town, thus adding want of food to the presence of heavy disease, but they absolutely refused entrance into their houses to messengers from town—in some instances, not permitting them even to cross the fence some distance from the house, keeping them to the road under the sight of well-loaded rifle-guns. There was disease in Knoxville, and the people of the country but exercised a proper prudence in not entering the town; but nothing less than the demoralizing, inhumanizing panic of contagion would have made them deny food to the hungry.

Beyond all doubt there are diseases that are transmissible by the way of contagion, but thus far, or at the present day, they are but few in number and but rarely encountered under circumstances to produce panic. Magendie, in 1838, said in France, "the law recognizes five diseases to be contagious; out of these five diseases, four ought to be erased from the lists."

Of course, when a case of contagious disease does present, it ought to be disposed of in such a manner as will best protect from its greater dissemination. But it is not from these known contagious diseases that alarm is occasioned in communities, but from those rapidly wide spreading ailments, ordinarily accompanied by large mortality, originating from causes having their source at the localities that are troubled, or " being universal in their prevalence their cause is so likewise," which are ascribed to contagion. Almost uniformly the people of the afflicted localities find the cause produced anywhere than just there, where, in truth in a majority of instances, but little search would develop conditions and circumstances abundantly sufficient to occasion the sickness. " In all ages, men have exhibited a marked reluctance to ascribe such diseases to causes of a local or domestic nature." Continuing to employ the language of the good La Roche—" in the minds of the public at large, who seldom, if ever, posses the requisite qualifications, or devote the time necessary for investigations of the sort, it is most natural to attribute wide-spreading diseases to an exotic origin, and their propagation to the effect of exposure, with or without contact of the well to the sick or to fomites. Discovering nothing palpable in an infected spot to which they can refer the development of these; observing no difference in external agencies as regards meteorological phenomena, and the condition of localities between healthy and unhealthy seasons; seeing the disease start up almost suddenly and spread rapidly; knowing at the same time that it prevails elsewhere, and also that vessels or individuals have arrived from places so visited, they can not avoid the conclusion that when it shows itself among them, it is necessarily the offspring of importation from contaminated localities. Nor can they perceive how a disease which spreads widely and quickly, which attacks persons who have had communication with the sick, and is, withal, as they think, imported, can do so otherwise than through the influence of personal contagion. Dr. Rush says, " the idea is produced by a single act of the mind; it requires neither comparison nor reasoning to adopt it, and therefore accords with the natural indolence of man. It moreover flatters his avarice and pride by throwing the origin of a mortal disease from his property and country. The principle of thus refering the origin of the evils of life from ourselves to others is universal. It began in Paradise, and has ever since been an essential feature in the character of our species." Nor is the doctrine less acceptable to physicians themselves, particularly at the outset of epidemical visitations, when the facts have not been sufficiently sifted, and minds are engrossed with the existing calamity. Some, doubtless, adopt those views after mature examination and careful study, but in many, if not most instances they are adhered to, because they save a vast deal of trouble, or appear to explain at once and in the simplest manner all the circumstances that occur. To investigate the causes of disease, to collect and compare discordant and often obscure facts, and to draw correct deductions therefrom, and sift the grain from the chaff, is not so agreeable and easy a matter as to be undertaken and to be effected in a satisfactory manner by practitioners of medicine generally, and is beyond the scope of the public at large. Some of the former are too indolent to undertake it, others are ill prepared, by proper mental training, or too much occupied to devote the necessary time to the investigation, and they all, therefore, rest satisfied with, or care not to reach beyond the views entertained by the unprofessional portion of the community. These views become, at last, favorite objects of general belief, and there happens in regard to them what Locke, long ago, said of others of a different nature: "Men espouse the well-endowed opinions in fashion, and then seek arguments either to make good their beauty or garnish their deformity. To this we must attribute the fact, that all wide-spreading maladies and, indeed, often diseases of more restricted prevalence, have at times been considered as the product of importation, and as being endowed with contagious properties."

